# Taxonomic revision and phylogenetic position  of *Osteocephalus festae* (Anura, Hylidae) with description of its larva

**DOI:** 10.3897/zookeys.70.765

**Published:** 2010-11-29

**Authors:** Santiago R. Ron, Eduardo Toral, Pablo J. Venegas, Charles W. Barnes

**Affiliations:** 1 Museo de Zoología, Escuela de Biología, Pontificia Universidad Católica del Ecuador, Av. 12 de Octubre y Roca, Aptdo. 17-01-2184, Quito, Ecuador; 2 División de Herpetología-Centro de Ornitología y Biodiversidad (CORBIDI), Santa Rita N˚105 Of. 202, Urb. Huertos de San Antonio, Surco, Lima, Perú

**Keywords:** Andes, Amazon, Anura, morphology, Phylogeny, *Osteocephalus buckleyi*, *Osteocephalus festae*, *Osteocephalus verruciger*, tadpole

## Abstract

Osteocephalus festae is an Amazonian species recently resurrected from a synonymy with Osteocephalus buckleyi. Because few specimens are known, its morphological variation, diagnostic characters, and distribution are poorly understood. Herein we determine its phylogenetic relationships and provide a complete taxonomic account based on recently collected specimens (adults and larvae) from nine localities in Ecuador and Peru. Osteocephalus festae is most similar to Osteocephalus verruciger from which it differs in having less tuberculate dorsal skin on males, smaller tympanum, and more tooth rows in the oral disk of larvae. A phylogeny based on mitochondrial DNA sequences, genes 12S and ND1, shows that Osteocephalus festae is closely related to Osteocephalus buckleyi, Osteocephalus mutabor and Osteocephalus verruciger. A clade consisting of Osteocephalus festae, Osteocephalus verruciger, and Osteocephalus buckleyi is characterized by stream dwelling tadpoles. Surprisingly, we found paraphyly among Ecuadorian populations of Osteocephalus buckleyi and Osteocephalus verruciger. The causes for paraphyly are unknown but in Osteocephalus buckleyi may result from the existence of cryptic species.

## Introduction

Osteocephalus is a genus of hylinae frogs (tribe Lophiohylini) distributed in the Amazon Basin and the Guiana Shield (Faivovich et al. 2005). There are 24 recognized species of which half have been resurrected or described since 2000 (Frost 2010). Despite these efforts, taxonomic problems persist, including undescribed species and binomials of unknown validity or poorly understood boundaries. One such case is Osteocephalus festae, a species described by Peracca (1904) on the basis of a single specimen.

The holotype of Osteocephalus festae is an adult female collected at “Valle Santiago”, Provincia Morona Santiago, Ecuador. After its description, this binomial was largely ignored until Trueb and Duellman (1971) synonymized it under Osteocephalus buckleyi (Boulenger, 1882) based on comparisons of the holotype of Osteocephalus festae with series of Osteocephalus buckleyi from Guyana, Colombia, Ecuador and Peru. This synonymy was followed by all systematic accounts until Jungfer (2010) correctly resurrected Osteocephalus festae on the basis of the distinctiveness between the holotype of Osteocephalus festae and Osteocephalus buckleyi. Jungfer (2010) also ascribed to Osteocephalus festae five specimens from Napo and Sucumbíos provinces, Ecuador.

**Figure 1.**
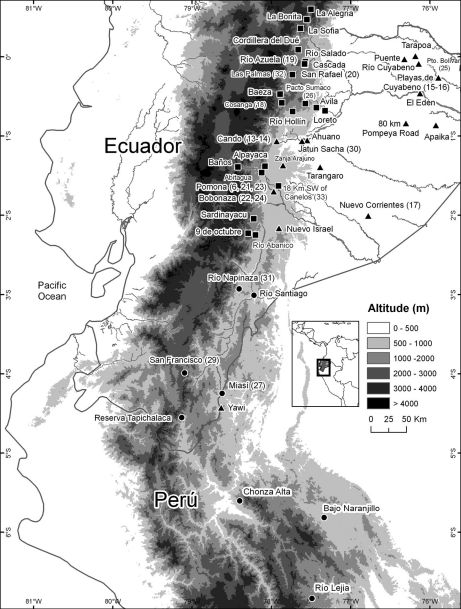
Records of Osteocephalus festae (circles), Osteocephalus verruciger (squares), and Osteocephalus buckleyi (triangles). Locality data from Trueb and Duellman (1970) and specimens deposited at the Museo de Zoología of Pontificia Universidad Católica del Ecuador, the Herpetology Collection, Escuela Politécnica Nacional, and CORBIDI (Appendix 1). Numbers correspond to those on Table 1 and Figure 2.

Recently collected specimens of Osteocephalus from nine populations from southeastern Ecuador and northeastern Peru, one of them at a distance of ~30 km from the type locality (Fig. 1), closely resemble the holotype of Osteocephalus festae and are morphologically and genetically distinctive from other species. They also seem to be distinctive from the specimens ascribed to Osteocephalus festae by Jungfer (2010) which may belong to a different species (see Taxonomic Remarks). Because little is known about Osteocephalus festae beyond the description of its holotype, below we provide an account of its variation, diagnosis, and distribution, as well as a description of its larvae. In addition, we assess its phylogenetic relationships using mitochondrial DNA sequences.

## Methods

### DNA extraction, amplification, and sequencing

Total DNA was extracted from muscle or liver tissue preserved in 95% ethanol and tissue storage buffer using a guanidine tiocyanate protocol. Polymerase chain reaction (PCR) was used to amplify the mitochondrial genes 12S rRNA and ND1. We amplified one DNA fragment for 12S and one or two overlapping fragments for ND1 using primers listed in Goebel et al. (1999) and Moen and Wiens (2009). PCR amplification was carried out under standard protocols. Amplified products were sequenced by the Macrogen Sequencing Team (Macrogen Inc., Seoul, Korea).

### Phylogenetic analyses

A list of the samples included in the phylogenetic analyses is shown in Table 1. For the outgroup, we included sequences of Osteopilus and Hypsiboas obtained from GenBank (http://www.ncbi.nlm.nih.gov/genbank). Outgroup choice was based on phylogenies showing that Osteocephalus is most closely related to Tepuihyla and Osteopilus (Faivovich et al. 2005 and Wiens et al. 2010). Because missing sequence data can result in misleading estimates of topology and branch lengths in phylogenies (Lemmon et al. 2009), we only included GenBank sequences for which both genes were availabel. Preliminary sequence alignment was done with CLUSTALW 1.83 (Chenna et al. 2003). The sequence matrix was imported to Mesquite (version 2.72; Maddison and Maddison 2009) and the ambiguously aligned regions were adjusted manually to produce a parsimonious alignment (i.e., informative sites minimized). Phylogenetic trees were obtained using Bayesian inference. The models of character evolution for the Bayesian analyses were chosen using JModelTest version 0.1.1 (Posada 2008) using the Akaike Information Criterion with sample size correction as optimality measure. We applied independent models to each of four partitions: one for 12S and three for each codon position in ND1. Four Markov chains were utilized in each of two Bayesian analyses, the prior for the rate matrix was a uniform dirichlet and all topologies were equally probable a priori. Each analysis ran for 5 × 106 generations. For each analysis, the chain was sampled every 1000 generations. After 5 x 106 generations the average standard deviation of split frequencies was ~ 0.002 indicating that the two analyses have converged into a stationary distribution. The first 50% of sampled trees were discarded as the burn-in and the remaining trees were used for estimating the Bayesian tree, posterior probabilities and other model parameters. Phylogenetic analyses were carried out in MrBayes 3.1.2 (Ronquist and Huelsenbeck 2003).

**Table 1.** Specimens used in the phylogenetic analysis. Numbers correspond to those in the Figures 1 and 2.

Museum No.	Species	No.	Genbank Accession No.		Reference
							12S	ND1
SBH 266458	Hypsiboas heilprini		DQ380357	EU034080	Moen and Wiens 2009; Wiens et al. 2006
USNM 327241	Osteopilus brunneus		DQ380382	EU034083	Moen and Wiens 2009; Wiens et al. 2006
SBH 266457	Osteopilus marianae		DQ380383	EU034086	Moen and Wiens 2009; Wiens et al. 2006
SBH 191985	Osteopilus pulchrilineatus		AY819436	EU034087	Moen and Wiens 2009; Wiens et al. 2005
QCAZ 15981	Osteocephalus alboguttatus	1	HQ600629	HQ600596	This study
LAC 2216	Osteocephalus buckleyi	30	DQ380378	EU034082	Moen and Wiens 2009; Wiens et al. 2006
QCAZ 14947	Osteocephalus buckleyi	17	HQ600628	HQ600595	This study
QCAZ 24446	Osteocephalus buckleyi	13	HQ600633	HQ600600	This study
QCAZ 24447	Osteocephalus buckleyi	14	HQ600634	HQ600601	This study
QCAZ 28231	Osteocephalus buckleyi	25	HQ600654	HQ600621	This study
QCAZ 28277	Osteocephalus buckleyi	15	HQ600639	HQ600606	This study
QCAZ 28395	Osteocephalus buckleyi	16	HQ600640	HQ600607	This study
QCAZ 32506	Osteocephalus buckleyi	22	HQ600651	HQ600618	This study
QCAZ 32508	Osteocephalus buckleyi	24	HQ600652	HQ600619	This study
QCAZ 37175	Osteocephalus buckleyi	23	HQ600653	HQ600620	This study
QCAZ 25469	Osteocephalus buckleyi	21	HQ600650	HQ600617	This study
CORBIDI 623	Osteocephalus festae	28	HQ600649	HQ600616	This study
QCAZ 38420	Osteocephalus festae	31	HQ600646	HQ600613	This study
QCAZ 39364	Osteocephalus festae	29	HQ600648	HQ600615	This study
QCAZ 41039	Osteocephalus festae	27	HQ600647	HQ600614	This study
QCAZ 20785	Osteocephalus fuscifacies	4	HQ600631	HQ600598	This study
QCAZ 25603	Osteocephalus mutabor	6	HQ600631	HQ600598	This study
QCAZ 28223	Osteocephalus mutabor	12	HQ600638	HQ600605	This study
QCAZ 28646	Osteocephalus mutabor	7	HQ600641	HQ600608	This study
QCAZ 28647	Osteocephalus mutabor	8	HQ600642	HQ600609	This study
QCAZ 30926	Osteocephalus mutabor	11	HQ600643	HQ600610	This study
QCAZ 40253	Osteocephalus mutabor	10	HQ600644	HQ600611	This study
QCAZ 42999	Osteocephalus mutabor	9	HQ600645	HQ600612	This study
QCAZ 20797	Osteocephalus planiceps	3	HQ600632	HQ600599	This study
QCAZ 18230	Osteocephalus taurinus	2	HQ600630	HQ600597	This study
QCAZ 15942	Osteocephalus verruciger	18	HQ600659	HQ600626	This study
QCAZ 15991	Osteocephalus verruciger	19	HQ600656	HQ600623	This study
QCAZ 20544	Osteocephalus verruciger	33	HQ600655	HQ600622	This study
QCAZ 32032	Osteocephalus verruciger	20	HQ600658	HQ600625	This study
QCAZ 41108	Osteocephalus verruciger	26	HQ600660	HQ600627	This study
QCAZ 27816	Osteocephalus yasuni	34	HQ600636	HQ600603	This study
QCAZ 27998	Osteocephalus yasuni	5	HQ600637	HQ600604	This study

### Morphological analyses

For ease of comparison, we generally follow the format of Trueb and Duellman (1971) for diagnosis and description. Morphological terminology and abbreviations follow Lynch and Duellman (1997) for adults and Altig and McDiarmid (1999) for tadpoles. Description of oral disk structure follows Altig and McDiarmid (1999). Notation for hand and foot webbing is based on Myers and Duellman (1982). Sex and reproductive condition was determined by the presence of nuptial pads, vocal sac folds, dorsal skin texture, and/or by gonadal inspection. Tadpoles were staged according to Gosner (1960) and preserved in 10% formalin. Other specimens were fixed in 10% formalin and preserved in 70% ethanol. To identify the tadpoles and juveniles we grew several tadpoles in captivity until they reached the juvenile stage. Juveniles exhibited a color pattern characteristic of Osteocephalus. The only other Osteocephalus known at the Río Napinaza collection site breeds on ponds (Osteocephalus taurinus) and has a different juvenile morphology (Lima et al. 2006).

Examined specimens (listed in the type-series and Appendix I) are housed at Museo de Zoología, Pontificia Universidad Católica del Ecuador (QCAZ), the Herpetology Collection, Escuela Politécnica Nacional (EPN-H), and the collection of the División de Herpetología, Centro de Ornitología y Biodiversidad (CORBIDI).

Principal Components Analysis (PCA) and Discriminant Function Analysis (DFA) were used to assess the degree of morphometric differentiation between adult Osteocephalus buckleyi, Osteocephalus festae, and Osteocephalus verruciger (Werner 1901). Only well preserved specimens (Simmons 2002) were measured for the following eight morphological variables, following Duellman (1970): (1) Snout-vent length (SVL); (2) head length; (3) head width; (4) tympanum diameter; (5) femur length; (6) tibia length; (7) foot length; and (8) eye diameter. All variables were log-transformed. To remove the effect of co-variation with SVL, the PCA and DFA were applied to the residuals from the linear regressions between the seven measured variables and SVL. We applied a multivariate analysis of variance (MANOVA) to test for morphometric differences between sexes. Because we found significant differences in Osteocephalus buckleyi, the PCA and DFA were applied to each sex separately. For the PCA, only components with eigenvalues > 1 were retained. Sample sizes for Osteocephalus verruciger were 23 males, 5 females; Osteocephalus festae 7 males, 18 females; and Osteocephalus buckleyi 25 males, 3 females. Both PCA and DFA were conducted in JMP® 8.01 (SAS Institute 2008).

Twelve morphometric variables were measured in tadpoles, following Altig and McDiarmid (1999): (1) total length; (2) body length; (3) body width; (4) body height; (5) tail length; (6) eye diameter; (7) oral disc width; (8) interorbital distance; (9) internarial distance; (10) maximum tail height; (11) tail muscle height; (12) tail muscle width. All measurements (adults and tadpoles) were made using digital calipers (to the nearest 0.01 mm). Larval tooth row formula is abbreviated as LTRF throughout.

## Results

### Phylogenetic relationships

**Figure 2.**
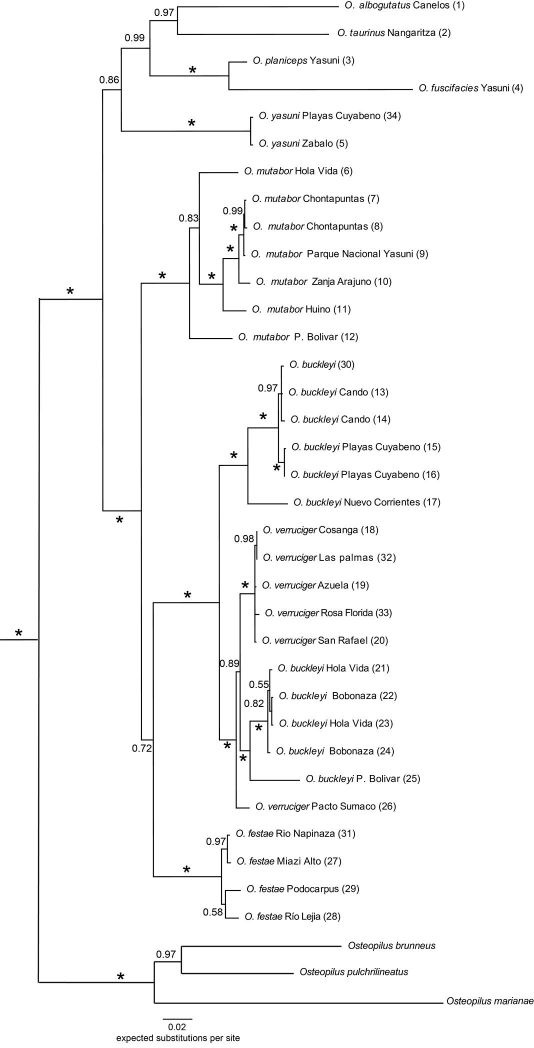
Bayesian consensus phylogram depicting relationships within Osteocephalus. Phylogram derived from analysis of 1975 bp of mtDNA (genes ND1 and 12S). Numbers in parenthesis corresponds to those on Table 1 and Figure 1. Posterior probabilities resulting from Bayesian Markov chain Monte Carlo searches appear above branches. An asterisk represents a value of 1. The outgroup species Hypsiboas heilprini is not shown.

The models with the best fit and the estimated parameters for each of four partitions for the Bayesian analyses are shown in Table 2. The Bayesian analyses of 1975 characters (1152 bp of ND1, 823 bp of 12S) resulted in a consensus tree showing strong support for an Osteocephalus clade (Fig. 2). Within Osteocephalus two clades have strong support: (1) Osteocephalus buckleyi, Osteocephalus festae, Osteocephalus mutabor, and Osteocephalus verruciger, and (2) Osteocephalus alboguttatus, Osteocephalus taurinus, Osteocephalus fuscifacies and Osteocephalus planiceps. A clade excluding Osteocephalus mutabor from (1) is weakly supported (posterior probability = 0.72).

**Table 2.** Post burn-in averages for parameters of Bayesian analyses. Abbreviations are: **AIC** = Akaike information criterion, **I** = proportion of invariant sites, **G** = shape parameter of the gamma distribution of rate variation, **k** = ratio of transition/transversion rates.

						Rate Matrix						Base Frequency			
Partition	Best-fit model	AIC score	I	G	K	AC	AG	AT	CG	CT	GT	A	G	C	T
12S	GTR + G + I	15762	0.386	0.740	-	0.079	0.281	0.081	0.007	0.522	0.026	0.334	0.183	0.238	0.243
ND1, 1st position	HKY + G + I	14527	0.489	1.385	14.22	-	-	-	-	-	-	0.365	0.129	0.240	0.264
ND1, 2nd position	GTR + G	6472	-	0.185	-	0.024	0.589	0.047	0.042	0.274	0.020	0.261	0.096	0.307	0.334
ND1, 3rd position	HKY + G + I	3745	0.295	1.617	14.22	-	-	-	-	-	-	0.397	0.104	0.216	0.280

Phylogenetic analysis shows that both Osteocephalus buckleyi and Osteocephalus verruciger are paraphyletic relative to each other. In Osteocephalus verruciger, the population of Pacto Sumaco is more closely related to one of two clades of Osteocephalus buckleyi than to the other populations of Osteocephalus verruciger. Seven populations of Osteocephalus buckleyi are separated in two well-supported clades, one of which is embedded within Osteocephalus verruciger. In contrast, monophyly among populations of Osteocephalus mutabor and Osteocephalus festae is strongly supported. Within Osteocephalus festae, pairwise uncorrected *p*-genetic distances range from 0.001 (Río Napinaza vs. Miasí) to 0.014 (San Francisco vs. Río Lejía). Uncorrected *p*-distances between both clades of Osteocephalus buckleyi range from 0.047 to 0.060. Distances between Pacto Sumaco and the other populations of Osteocephalus verruciger range from 0.015 to 0.018.

### Systematic account of Osteocephalus festae

#### Osteocephalus festae

(Peracca, 1904)

**Figure 3.**
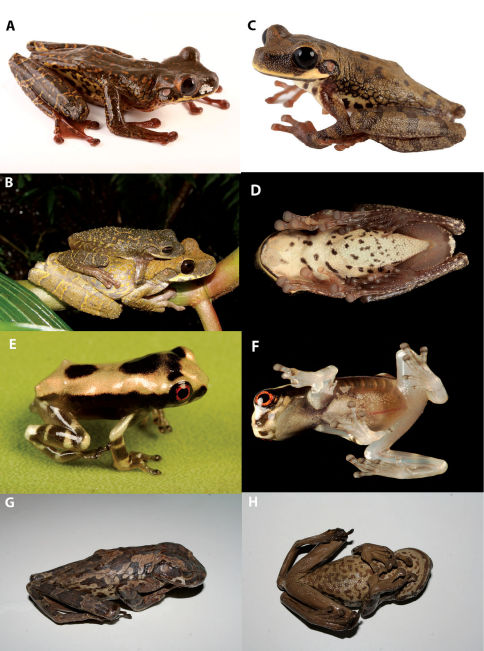
Dorsolateral and ventral views of Osteocephalus festae. **A** QCAZ 41039, adult female, SVL = 79.81 mm **B** Amplectant pair (not collected) from Chonza Alta, Peru **C–D** QCAZ 45674, subadult, SVL = 36.54 mm **E–F** QCAZ 38081, juvenile, SVL = 13.37 mm **G–H** MZUT An208 (holotype), adult female, (SVL = 78.00 mm; Peracca 1904). Holotype photographs by Franco Andreone and photographs of the amplectant pair by PJV. See Appendix I for locality data.

Hyla festae  Peracca, 1904:39. Holotype: MZUT An208, a female from “Valle Santiago” (“= lower Río Zamora” according to Trueb and Duellman 1971) Provincia Morona Santiago, Ecuador (Fig. 3G-H).
Osteocephalus buckleyi  (part) Trueb and Duellman 1971. Synonymy fide Trueb and Duellman 1971: 23.

##### Diagnosis.

**Figure 4.**
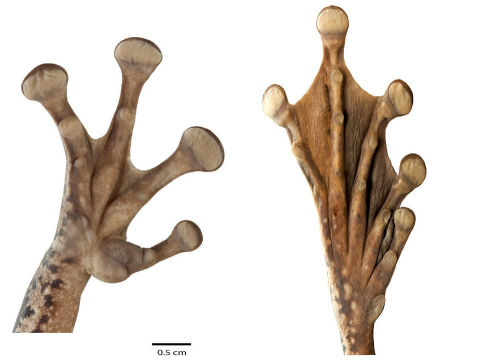
Ventral views of the right hand and foot of Osteocephalus festae. Adult female from Río Napinaza, Ecuador, SVL = 84.93 mm, QCAZ 39811. Hand and foot are shown at the same scale.

**Figure 5.**
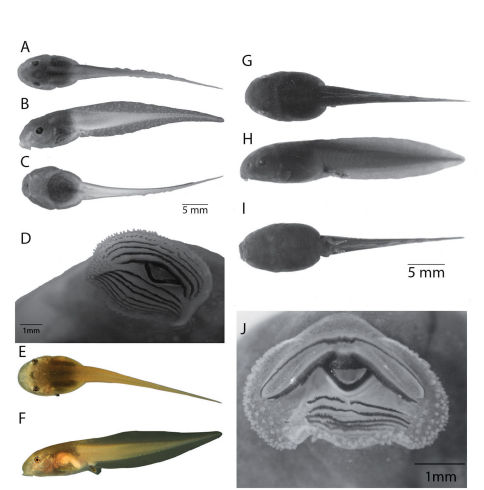
Tadpoles of Osteocephalus festae and Osteocephalus verruciger. A–D, G–J are in preservative; E–F in life. A–D: Osteocephalus festae, stage 39, QCAZ 30511; E–F: Osteocephalus festae, stage 33, QCAZ 38074; G–J: Osteocephalus verruciger,stage 36, QCAZ 36751. A, G: dorsal view; B, H: lateral view; C, I: ventral view; D, J:oral apparatus. Photos in life by SRR.

Throughout this section, coloration refers to preserved specimens unless otherwise noted. Osteocephalus festae is a medium-sized species of Osteocephalus having the following combination of characters : (1) size sexually dimorphic; maximum SVL in males 56.1 mm, in females 84.9 mm; (2) skin on dorsum bearing tubercles in males, smooth in females; (3) skin on flanks areolate; (4) hand webbing formula **II**2½—3¼**III**3½—2**IV**; foot webbing formula varying as shown in Table 3 and Fig. 4; (5) dorsum brown, usually with irregular dark marks; (6) venter varying from cream to tan, with ill to well defined brown chocolate blotches; (7) narrow, cream to light brown, labial stripe confluent with similarly colored suborbital mark; (8) flanks cream to light brown with darker reticulations anteriorly and dark blotches posteriorly; (9) dermal roofing bones of the skull weakly exostosed; (10) bones green in life; (11) iris dark brown without reticulations, in life; (12) paired vocal sacs located laterally, behind jaw articulation, (13) in life, juveniles with red iris, and pale elbows, knees, and heels; (14) larvae with LTRF of 4/7 or 5/7.

Osteocephalus festae is most similar to Osteocephalus verruciger. Both species differ from other Osteocephalus by the combination of a brown iris (in life) and the presence of brown marks in the venter (in life and preservative). Osteocephalus festae differs from Osteocephalus verruciger in having: (1) less dorsal ornamentation in males (fewer and less developed dorsal tubercles in Osteocephalus festae), (2) smaller tympanum (1/5 of head length in Osteocephalus festae vs. 1/4 in Osteocephalus verruciger), and (3) more tooth rows in larvae (LTRF = 4/7 to 5/7 in Osteocephalus festae vs. 2/5 in Osteocephalus verruciger; Fig. 5). Mitochondrial DNA sequences show that Osteocephalus festae and Osteocephalus verruciger are not sister species (Fig. 2). Osteocephalus festae differs from Osteocephalus mutabor Jungfer & Hödl, 2002 in having chocolate blotches in the venter (blotches absent in Osteocephalus mutabor). It differs from most species of Osteocephalus (except Osteocephalus verruciger, Osteocephalus heyeri Lynch, 2002, and Osteocephalus subtilis Martins & Cardoso, 1987) in having a dark brown iris in life (iris bronze to golden with or without black straight lines or irregular black reticulations in Osteocephalus alboguttatus (Boulenger, 1882), Osteocephalus buckleyi, Osteocephalus cabrerai (Cochran & Goin, 1970), Osteocephalus deridens Jungfer et al., 2000, Osteocephalus exophthalmus Smith & Noonan, 2001, Osteocephalus fuscifacies Jungfer et al., 2000, Osteocephalus leoniae Jungfer & Lehr, 2001, Osteocephalus leprieurii (Duméril & Bibron, 1841), Osteocephalus mutabor, Osteocephalus oophagus Jungfer & Schiesari, 1995, Osteocephalus pearsoni (Gaige, 1929), Osteocephalus phasmatus MacCulloch & Lathrop, 2005, Osteocephalus planiceps Cope, 1874, Osteocephalus taurinus Steindachner, 1862, and Osteocephalus yasuni Ron & Pramuk, 1999). Osteocephalus mimeticus (Melin, 1941) can be discriminated from Osteocephalus festae in having a black iris with golden marks. Osteocephalus festae differs from Osteocephalus subtilis and Osteocephalus heyeri in size (maximum male SVL 38.8 mm in Osteocephalus subtilis, 36.1 mm in Osteocephalus heyeri vs. 56.1 mm in Osteocephalus festae). The presence of areolate skin in the flanks, specially anteriorly, distinguish Osteocephalus festae from Osteocephalus leprieurii, Osteocephalus mutabor, Osteocephalus pearsoni, Osteocephalus planiceps, and Osteocephalus yasuni (smooth to granular skin on flanks; Trueb & Duellman, 1971). Osteocephalus taurinus has weakly areolate skin restricted to the axillary region. Osteocephalus festae further differs from Osteocephalus buckleyi and Osteocephalus cabrerai in lacking prominent tarsal tubercles (Jungfer 2010).

##### Holotype.

The holotype is an adult female with SVL = 78 mm (Fig. 3G, H; Peracca, 1904). The descriptions provided by Peracca (1904) and Jungfer (2010) are adequate.

##### Variation.

**Figure 6.**
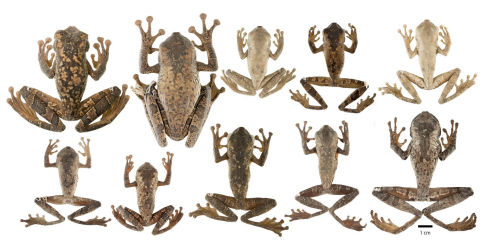
Adult Osteocephalus festae showing variation in dorsal coloration of preserved specimens. Left to right, upper row: QCAZ 39804, 39811, 39810, 39802, 39798 (females); lower row: QCAZ 39799, 26552, 26488, 26561 (males), 39364 (female). Provincia Loja and Morona Santiago, Ecuador. See Appendix I for locality data.

**Figure 7.**
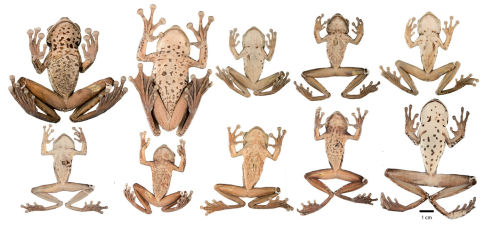
Ventral views of adult Osteocephalus festae showing variation in ventral coloration of preserved specimens. Specimen identity and arrangement is the same as in Figure 6.

Variation in dorsal and ventral coloration of preserved specimens is shown in Figures 6 and 7. Dorsal coloration consists of a light brown to dark brown background with irregular marks. There is sexual dimorphism in dorsal tuberculation: in females the dorsum is smooth while in males it varies between having scant and ill-defined non-keratinized tubercles (most males from Río Napinaza, e.g., QCAZ 26488) to having abundant keratinized tubercles (two males from Chonza Alta, e.g., CORBIDI 758; Fig. 3B).

Ventral surfaces of preserved specimens (Fig. 7) have a cream (QCAZ 39364) to tan (QCAZ 39806) background with darker brown marks that are more distinct and abundant in females (e.g., QCAZ 39811) than in males (e.g., QCAZ 39799); a male from Río Lejía (CORBIDI 623) has an immaculate venter. Ventrally, limbs vary from brown to cream; in QCAZ 39809 and 39811 cream dots are present on hindlimbs; scant cream tubercles can be present in the external edge of the forearm (e.g., QCAZ 39804). The vent region is dark brown to brown bordered by a lighter area (cream to tan). Flanks are areolate in the anterior half and smooth posteriorly. The areolate portion is cream with dark brown reticulation; the posterior half is cream (e.g., QCAZ39810) to light brown (e.g., QCAZ 39806) with dark brown blotches.

Head shape is rounded in dorsal view and rounded (e.g., QCAZ 39803) to bluntly rounded (e.g., QCAZ 39800–01) in lateral view. Lateral head coloration varies between dark brown (QCAZ 11625) to light brown (QCAZ 39810). Except for QCAZ 39802 and 41039, there is a lighter (brown to cream) subocular mark. A tan (QCAZ 39805) to cream (QCAZ 39364) labial stripe is always present. The tympanic annulus is concealed dorsally and has lighter color than the background. Variation in hand and foot webbing is shown in Table 3. The distal subarticular tubercle on Finger IV is single in all specimens.

**Table 3.** Variation in webbing in hand and feet of representative adults of Osteocephalus festae. Webbing formula notations follow Savage and Heyer (1967) with modifications by Myers and Duellman (1982).

	Hand	Foot
QCAZ 39805 (female)	I basal II2½—3¼III3½—2+IV	I1–—1–II1–—1–III1–—1–IV1–—1–V
QCAZ 39809 (female)	I basal II2½—3¼III3½—2+IV	I1–—1–II1–—2–III1–—2½IV2½—1–V
QCAZ 39799 (male)	I basal II2½—3¼III3½—2+IV	I1—1II1—1–III1–—2½IV2½—1–V
QCAZ 39802 (female)	I basal II2½—3¼III3½—2+IV	I1+—2–II1+—2III1—2IV2—1–V
QCAZ 26304 (female)	I basal II2½—3¼III3½—2–IV	I1–—1–II1–—1–III1–—1–IV1–—1–V
QCAZ 26488 (male)	I basal II2½—3¼III3½—2–IV	I1–—1–II1–—1–III1–—1–IV1–—1–V

Morphometric data pertain to adults and are summarized in Table 4. In the examined series, the largest male has a SVL of 56.09 mm and the largest female 84.94 mm; mean male SVL = 49.47 mm (*n* = 12; SD = 5.10), mean female SVL = 67.92 mm (*n* = 27; SD = 10.08). Females are significantly larger than males (*t* = 5.52, df = 23, *P* < 0.001). A MANOVA on the residuals of the regressions between SVL and the other measured variables indicates lack of significant differences between sexes in size-free morphometry (*F* = 1.052, df = 17, *P* = 0.433).

**Table 4.** Descriptive statistics for morphometric measurements of adult Osteocephalus festae. Mean ± SD is given with range below. Bold figures are averages for individuals of all populations. Abbreviations are: **SVL** = snout-vent length; **FOOT** = Foot Length; **HL** = Head Length; **HW** = Head Width; **ED** = Eye Diameter; **TD** = Tympanum Diameter; **TL** = Tibia Length; **FL** = Femur Length. All measurements are in mm.

Species	SVL	FOOT	HL	HW	ED	TD	TL	FL
Males (n = 12)	47.47 ±	19.38 ±	14.51 ±	15.77 ±	5.01 ±	2.79 ±	25.90 ±	22 ±
Females (n = 27)	67.91 ±	29.79 ±	20.31 ±	22.60 ±	6.25 ±	3.72 ±	39.13 ±	35.35 ±
Chonza Alta								
Males (n = 4)	43.72 ±	17.97 ±	14.07 ±	13.82 ±	4.51 ±	2.55 ±	24.03 ±	22.05 ±
Females (n = 8)	68.72 ±	29.33 ±	20.62 ±	22.33 ±	6.2 ±	3.26 ±	39.56 ±	34.63 ±
Miasí								
Female (n = 1)	79.81	36.65	21.93	26.12	6.98	3.8	45.73	42.19
Río Lejia								
Males (n = 1)	48.3	20.7	14.3	16.8	4.3	2.8	26.8	24.6
Females (n = 1)	72.6	30	21.1	23.2	6.1	3.7	41.2	30.4
Río Napinaza								
Males (n = 4)	50.82 ±	21.08 ±	14.62 ±	17.4 ±	5.66 ±	2.96 ±	27.67 ±	24.88 ±
Females (n = 16)	66.37 ±	29.59 ±	20.04 ±	22.53 ±	6.28 ±	3.96 ±	38.35 ±	35.55 ±
San Francisco								
Female (n = 1)	69.54	29.48	19.63	21.73	5.72	3.55	39.63	35.90
San Carlos								
Males (n = 3)	47.74 ±	19.31 ±	15.00 ±	15.86 ±	5.04 ±	2.87 ±	25.72 ±	23.23 ±

##### Color in life.

Based on digital photograph of adult female QCAZ 41039 (Fig. 3A): dorsum dark brown with irregular light brown and yellowish green marks; canthal region dark brown with yellowish green subocular mark and labial band; tympanum brown; flanks greenish brown with dark brown reticulation anteriorly and irregular dark brown marks posteriorly; dorsal surfaces of thighs and shanks dark brown with transversal brown bands bordered with light brown; dorsal surfaces of forelimbs dark brown with irregular brown marks; venter light tan with irregular brown marks; bones green; iris dark brown. Female CORBIDI 761 has a predominantly light brown dorsum with irregular brown marks; clear areas on flanks and below the eye and tympanum are light yellow.

There is significant change in coloration between juveniles and adults. The following description is based on a digital photograph of juvenile QCAZ 38081 (Fig. 3E-F). The dorsum beige with black interorbital band and two large medial ovoid black blotches; flanks dark brown; dorsal surfaces of thighs and shanks brown with cream transversal bars; dorsal surfaces of arms cream, dorsal surfaces of forearms brown with cream transversal bars; knees, elbows, and heels cream; anterior half of the venter cream, posterior half light brown; bones green; iris bright red.

##### Morphometric comparisons.

**Figure 8.**
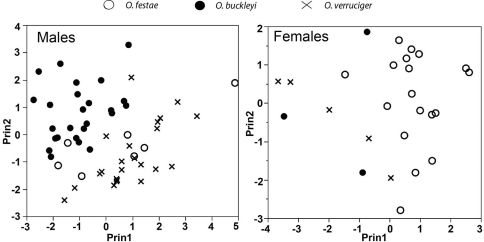
Principal components from analysis of seven size-corrected morphological variables. See Table 5 for character loadings on each component.

Three components with eigenvalues > 1.0 were extracted from the PCA for males (Table 5). The three components accounted for 76.4% of the total variation. The highest loadings for the PCA for males were foot length and tibia length for PC I, head length for PC II, and eye diameter and tympanum diameter for PC III (Table 5). The morphometric space of Osteocephalus festae overlaps with Osteocephalus verruciger (Fig. 8) but only slightly with Osteocephalus buckleyi. There are significant differences in PC scores between Osteocephalus festae and Osteocephalus buckleyi along PC II and PC III (*t* = 2.46, df = 9, *P* = 0.035; *t* = 6.76, df = 13, *P* < 0.001, respectively) but not along PC I (t = 1.61, df = 7, *P* = 0.115). There are significant differences only along PC III between Osteocephalus festae and Osteocephalus verruciger, (*t* = 9.03, df = 9, *P* < 0.001).

**Table 5.** Character loadings and eigenvalues for Principal Components (PC) I–III. The analysis was based seven morphometric variables of adult Osteocephalus. Bold figures indicate highest loadings.

	PCA Males			PCA Females		
Variable	PC I	PC II	PC III	PC I	PC II	PC III
Foot length	0.523	0.564	0.242	0.459	0.202	– 0.151
Head length	– 0.243	0.647	0.121	– 0.243	0.394	0.537
Head width	0.407	– 0.430	– 0.208	0.196	0.315	– 0.680
Eye diameter	– 0218	0.259	– 0.673	– 0.061	0.648	– 0.125
Tympanum diameter	– 0.224	0.004	0.655	– 0.435	0.451	– 0.016
Tibia length	0.455	0.407	– 0.020	0.519	0.096	0.285
Femur length	0.441	0.398	– 0.005	0.476	0.266	0.356
Eigenvalue	2.570	1.744	1.030	2.722	1.517	1.382

Three components with eigenvalues > 1.0 were extracted from the PCA for females (Table 5). The three components accounted for 80.3% of the total variation. The highest loadings for the PCA for females were tibia length and femur length for PC I, eye diameter and tympanum diameter for PC II, and head width and head length for PC III (Table 5). There is little overlap in the morphometric space of Osteocephalus festae with Osteocephalus verruciger and Osteocephalus buckleyi (Fig. 8). Principal Components scores are significantly different between Osteocephalus festae and Osteocephalus verruciger along PC I and PC III (*t* = 4.96, df = 21, *P* < 0.001; *t* = 4.91, df = 21, *P* < 0.001, respectively) but not along PC II (*t* = 0.85, df = 21, *P* = 0.403). Similarly, Osteocephalus festae and Osteocephalus buckleyi differed along PC I and PC III (*t* = 4.96, df = 21, *P* < 0.001; *t* = 4.91, df = 21, *P* < 0.001, respectively) but not along PC II (*t* = 0.85, df = 21, *P* = 0.403).

In the DFA classification procedure, 24 out of 25 specimens of Osteocephalus festae were classified correctly. The misclassified female (QCAZ 38420) was assigned to Osteocephalus verruciger. The multivariate analyses (PCA and DFA) show morphometric differentiation between Osteocephalus festae and the closely related Osteocephalus verruciger and Osteocephalus buckleyi.

##### Tadpoles.

Letters in parenthesis refer to individual tadpoles on each lot. The following description is based on lot QCAZ 30511 of ten larvae in Stages 25 (A), 26 (B), 31 (C), 32 (D), 33 (E), 34 (F), 35 (G), 39 (H), 40 (I) and 42 (J). Tadpoles were collected at Río Napinaza by E. E. Tapia and I. G. Tapia on October 2003. These larvae belong to the exotrophic, benthic guild as defined by McDiarmid and Altig (1999). Morphometric data are provided in Table 6. In dorsal view, a tadpole in Stage 39 (QCAZ 30511H; Fig. 5A) shows elliptical body, widest between eye and spiracle, with rounded snout. Eyes relatively large (body length about 7.89 times larger than eye diameter), directed and positioned dorsolaterally, not visible in ventral view, and separated by a distance 1.27 times the internarial distance. External nares oval, located dorsolaterally, at about one fourth the distance between anterior margin of snout and anterior margin of eye. In profile (Fig. 5B) body depressed (body width/body height = 0.18), flattened ventrally, snout slightly rounded. Oral disc not emarginated. Spiracle sinistral, inner wall free from body, its tip closer to the vent than the eye. Spiracle opening rounded.

**Table 6.** Measurements of tadpoles of Osteocephalus festae (lot QCAZ 30511). Developmental stages, in parentheses, are defined according to Gosner (1960). Abbreviations are: **TL** = Total Length; **BL** = Body Length; **BW** = Body Width; **BH** = Body Height; **TAL** = Tail Length; **ED** = Eye Diameter; **ODW** = Oral Disc Width; **IOD** = Interorbital Distance (measured between center pupils); **IND** = Internarial Distance (measured between centers of narinal openings); **MTH** = Maximum Tail Height; **TMH** = Tail Muscle Height; **TMW** = Tail Muscle Width. All measurements in mm.

	Stage									
Variable	A (25)	B (26)	C (31)	D (32)	E (33)	F (34)	G (35)	H (39)	I (40)	J (42)
TL	30.05	29.49	37.22	35.57	34.75	34.68	39.81	40.39	40.63	40.94
BL	9.51	9.19	11.06	10.07	10.82	11.04	11.44	11.13	11.81	11.16
BW	5.74	5.68	7.25	7.05	7.23	7.10	8.11	7.50	8.46	7.12
BH	4.83	4.98	5.95	5.46	5.87	5.88	6.10	6.32	6.27	4.89
TAL	20.30	20.04	26.59	24.56	24.36	23.76	27.90	28.99	28.69	29.90
ED	1.02	1.30	1.25	1.28	1.47	1.10	1.50	1.41	1.70	1.47
ODW	3.50	3.35	3.90	4.12	3.80	4.43	4.13	4.00	3.94	3.46
IOD	4.27	4.34	5.15	5.40	5.08	5.45	5.04	5.26	5.74	5.02
IND	3.49	3.50	4.01	4.19	3.75	3.94	3.98	4.12	4.01	1.62
MTH	5.30	5.37	5.88	6.10	5.85	6.05	6.70	6.62	6.75	6.06
TMH	2.06	2.00	2.57	2.50	3.26	2.79	3.07	3.07	3.45	2.44
TMW	1.60	1.46	2.70	2.12	2.30	2.50	2.97	2.62	2.78	2.56

Tail musculature robust, decreasing in size towards tip of tail. Dorsal fin not extending onto body, slightly convex and attaining its maximum height at mid length of tail; tail tip rounded; ventral fin convex, beginning at tail-body junction and tapering gradually towards tail tip. Medial vent tube with both walls attached directly to ventral fin, opening directed posteroventrally. Limbs with subarticular patches. Dorsal body, middle body, supraorbital, infraorbital, posterior supraorbital, and posterior infraorbital lateral lines evident. No glands.

Oral disc anteroventral (Fig. 5B; average transverse width 4.35 mm; 58% of body width), not emarginate, LTRF 4/7; papillae distributed around oral disc; tooth rows complete except for medial gap in row A4; A1 = 304, A2 = 322, A3 = 311, A4 = 108 + 112; P1 = 177, P2 = 235, P3 = 234, P4 = 328, P5 = 284, P6 = 330, and P7 = 177.

In preservative, dorsum brown with darker marks between eyes; dark brown dorsolateral stripes extend from mid-body to base of tail; caudal musculature beige with brown spots (Fig 5); skin of flanks, spiracle, vent tube, fins, and around the eyes transparent; belly and fins transparent with white blotches.

##### Tadpole variation and comparisons with other species.

In QCAZ 38074, 26321, 26053, 26498 and 26284 the caudal musculature is cream with brown dots; fins can have dark brown spots without white blotches (e.g., QCAZ 38074, 26321). The LTRF is the same in all stages but in stage 42, rows A2–A4 and P1–P3 have approximately half the number of teeth.

In preservative, ten tadpoles collected in Chonza Alta, Peru, lot CORBIDI-CL-10 in Stages 37 (A-B), 36 (C), 34 (D), 32 (E), 31 (F), 42 (G, H, I) and 44 (J) have dorsum brown with darker marks between the eyes; dark brown dorsolateral stripes from mid-body to base of tail; caudal musculature cream with abundant melanophores; skin of flanks, spiracle, vent tube, and fins transparent; skin around the eyes brown; belly and fins transparent with abundant melanophores. Oral disc with LTRF 5/7; papillae distributed around oral disc; tooth rows complete except for medial gap in row A5.

In life (Fig. 5E-F; QCAZ 38074, stage 33; based on digital photograph), dorsum dark brown with darker marks between the eyes; dark brown dorsolateral lines from mid-body to base of tail; tail musculature light brown with small dark brown melanophores; white dots at tail-body junction; skin is transparent ventrally in anterior half of body and ventrolaterally in the posterior half; tail musculature light brown with dark brown spots; fins transparent. Iris bronze. Live tadpoles from Chonza Alta (CORBIDI-CL-10) have dorsum and caudal musculature olive brown; skin transparent ventrally with bright brown flecks, gut visible through the skin; fins translucent brown.

Larvae of Osteocephalus verruciger differ from those of Osteocephalus festae (in parentheses) by being smaller, having uniform dark body (tan to cream), LTRF = 2/5 (4/7 to 5/7); and having a dorsal gap in marginal papillae (marginal papillae complete). For comparison, larvae of Osteocephalus verruciger is shown in Figure 5 G–H. Differences were verified in 30 Osteocephalus verruciger larvae from four localities (lots QCAZ 1579, 10798, 21405 and 36751). Larvae of Osteocephalus festae have the highest number of tooth rows known among Osteocephalus (LTRF = 2/3 in Osteocephalus oophagus, 2/6 in Osteocephalus buckleyi and Osteocephalus taurinus; Hero 1990; Jungfer and Schiesari 1995).

##### Distribution and ecology.

Osteocephalus festaehas been recorded at nine localities in the Ecuadorian (Loja, Morona Santiago, and Zamora-Chinchipe provinces) and Peruvian Amazon basin (Mariscal Cáceres and Rioja provinces). Localities with known elevation (Río Napinaza, Miasí, San Francisco, Reserva Tapichalaca, Río Lejia, Chonza Alta, Camñopite Bajo, and Naranjillo) range between 1000 and 2200 m of elevation. The elevation at San Francisco (2200 m) is the highest known for Osteocephalus. Maximum airline distance between localities is 440 km. Osteocephalus festae and Osteocephalus verruciger have similar elevational ranges and seem to replace each other latitudinally in Ecuador (Fig. 1). Records of Osteocephalus verruciger from Peru (e.g., Trueb and Duellman 1970) are likely misidentified Osteocephalus mimeticus (Jungfer 2010). Thus, the southernmost confirmed records of Osteocephalus verruciger are those from Provincia Morona Santiago, Ecuador.

Most of our specimens of Osteocephalus festae are from Río Napinaza, a river surrounded by secondary forest, pastures and agricultural lands. At the collection site, the river has an average width of 2.85 m and an average depth of 23 cm with fast running water and waterfalls that reach 10 m in height (Salazar-Valenzuela 2007). Tadpoles were found in small ponds in the margins of the river. Adults were observed at night next to the river or within the forest on vegetation 40 to 250 cm above the ground.

All the specimens collected in Las Cataratas de Paraiso (Chonza Alta) and Camñopite were found at night on vegetation 50 to 300 cm above the ground, next to fast running streams. Tadpoles (CORBIDI-CL-10) were found in a rocky stream with average width of 4 m and an average depth of 30 to 40 cm with fast running water, close to the base of a waterfall. At both sites the streams are surrounded by secondary forest, pastures and agricultural lands. The specimens from Bajo Naranjillo and Río Lejia were found at night on branches 150 to 200 cm above the ground (in primary forest at Río Lejia and secondary forest surrounded by pastures at Naranjillo).

At Las Cataratas de Paraiso (Chonza Alta), on 1 December 2007, we found twelve males calling from the low vegetation and six amplectant pairs (Fig. 3B). Recently metamorphosed individuals were perching on leaves and rocks at the shore. On 4 November 2008, at the same stream, we only found one adult non-amplectant female, two adult males, several tadpoles, and 12 freshly metamorphosed juveniles on leaves and rocks (e.g., CORBIDI 1962–64). The rainy season in this region generally starts in December but during our surveys heavy rains fell since the first week of November. Three gravid females (CORBIDI 761, SVL 68.6 mm; CORBIDI 762, SVL 69.1 mm; CORBIDI 764, SVL 66.6 mm) contained 1080, 1388 and 1290 eggs respectively. A gravid female CORBIDI 624 (SVL = 71.2 mm) from Río Lejia, collected on 29 January 2008, contained 780 eggs. The color of eggs in preservative is black and brownish-cream.

Vegetation types for Ecuadorian localities (according to the classification of Sierra et al. 1999 ) are: (1) Evergreen Foothill Forest of the Eastern Slopes of the Southern Andes, characterized by abundant epiphytes, trees reaching 30 m of height with Podocarpus as dominant species (Reserva Tapichalaca), (2) Amazonian Mountain Range Evergreen Foothill Forest, characterized by a mixture of Amazonian and Andean vegetation with a canopy of 30 m (Río Napinaza and Miasí), and (3) Cloud Montane Forest of the Eastern Slopes of the Southern Andes, characterized by trees covered by mosses and abundant epiphytes (San Francisco; Cerón et al. 1999).

Vegetation types of the Peruvian localities (according to Duellman and Pramuk 1999) are Humid Subtropical Forest, characterized by of a variety of moderate to large trees including Juglans neotropica, Cedrela fissipes, Tabebuia, and genera common in the Amazon lowlands like Brosimum, Cordia, Inga, Piper, and Swietenia. At some collecting sites, the forest has been cleared for citrus and coffee plantations.

##### Taxonomic remarks.

Jungfer (2010) ascribed two females (EPN 5578, MHNG 2560.60) and three males (EPN 5577, EPN AA-5611, EPN 5607) to Osteocephalus festae. Based on data from Jungfer (2010), both females differ from the holotype and 18 adult females analyzed here (in parentheses) in having a venter uniform tan without marks (brown marks present), webbing almost reaching the ultimate subarticular tubercle in the inner edge of third finger (web reaching half the distance between ultimate and penultimate subarticular tubercles), and larger tympanum size with TD/HL = 0.23–0.25 (TD/HL = 0.14–0.22 among 39 adult males and females). In addition, we did not find the sexual dimorphism in relative tympanum size reported by Jungfer (Student’s *t* = 1.227, df = 23, *P* = 0.232). Males assigned to Osteocephalus festae also seem to differ from our series in the extent of the axillary membrane (covering half of the upper arm vs. one third to one fourth in our series). The discrepancies suggest that at least some of the specimens assigned to Osteocephalus festae by Jungfer (2010) may belong to a different species.

## Discussion

The phylogenetic relationships recovered by this study are consistent with the phylogenies reported by Wiens et al. (2010) and Moen and Wiens (2009) in finding strong support for a clade that closely allies Osteocephalus buckleyi, Osteocephalus verruciger, and Osteocephalus mutabor. Our clade (Osteocephalus mutabor (Osteocephalus festae (Osteocephalus buckleyi-Osteocephalus verruciger))) is composed by species that mainly reproduce along streams or slow flowing ditches. At several sites we have found tadpoles of Osteocephalus verruciger in ponds on stream banks and also on slow flowing ditches confirming the riparian habits reported by Trueb and Duellman (1970). Osteocephalus buckleyi breed along streams (Jungfer 2010; Lima et al. 2006) and Osteocephalus mutabor has been found breeding along ditches (SRR pers. obs.; Jungfer and Hödl 2002) and temporary ponds (M. Read, pers. comm.) The predominance of stream breeding habits among these species suggests that reproductive mode may be phylogenetically conserved in Osteocephalus. The same pattern is suggested by the close relationship between Osteocephalus planiceps, Osteocephalus deridens, Osteocephalus fuscifacies implied by our phylogeny and that of Moravec et al. (2009) because these species share phytotelmata breeding (SRR pers. obs.; Jungfer et al. 2000). This reproductive mode, however, may have an additional independent origin in Osteocephalus oophagus (Moravec et al. 2009).

An unexpected result in our phylogeny is the finding of paraphyly among populations of Osteocephalus buckleyi and Osteocephalus verruciger from Ecuador. Plausible explanations include incomplete lineage sorting, mitochondrial gene capture, and the existence of cryptic species hidden within each taxa. In the case of Osteocephalus verruciger, the position in the phylogeny of the population that generates paraphyly, Pacto Sumaco, is weakly supported (Fig. 2) and the genetic distance between Pacto Sumaco and the other Osteocephalus verruciger populations is lower (1.5–1.8% sequence divergence) than the distances between Osteocephalus verruciger and Osteocephalus buckleyi or between any other species pair in the phylogeny (> 2.1%). In addition, we could not find conspicuous morphological differences between Pacto Sumaco and the other Osteocephalus verruciger populations. The observed pattern suggest that our mitochondrial gene tree may not correctly reflect the history of divergence among Osteocephalus verruciger and the morphologically distinctive populations of Osteocephalus buckleyi from Hola Vida and Bobonaza. Conversely, the paraphyly among populations of Osteocephalus buckleyi has a strong support with higher genetic distances between both clades (4.7%–6.0%). Neither of these clades represent Osteocephalus cabrerai, a species morphologically similar to Osteocephalus buckleyi for which there are not confirmed records from Ecuador (Jungfer 2010). Examination of additional characters (morphological and molecular) is underway by SRR to determine the taxonomic status of the populations of Osteocephalus buckleyi from western Amazonia.

## Supplementary Material

XML Treatment for Osteocephalus festae

## Appendix I

Osteocephalus buckleyi.— ECUADOR: PROVINCIA MORONA SANTIAGO: Bobonaza, 660 m (QCAZ 32506–08); PROVINCIA NAPO: Cando, 700 m (QCAZ 24446–47); Ahuano, 410 m (QCAZ 36703); Serena, Río Jatunyacu, 520 m (QCAZ 25321); PROVINCIA ORELLANA: Río Yasuní (QCAZ 7360); Puente del Río Beque, 228 m (QCAZ 43071); Río Rumiyacu, Parque Nacional Yasuní, 250 m (QCAZ 16007); Taracoa, 251 m (QCAZ 34963); Aguarico, Parque Nacional Yasuní (EPN-H 2786); PROVINCIA PASTAZA: Pomona, Fundación Hola Vida, 846 m (QCAZ 25469, 25607, 37175); Nuevo Corrientes, 250 m (QCAZ 14947); Tarangaro, 338 m (QCAZ 39073); Arajuno, Curaray, Río Maderoyacu (EPN-H 6374); PROVINCIA SUCUMBÍOS: Tarapoa (QCAZ 14948); Puerto Bolívar, 240 m (QCAZ 28231); Playas de Cuyabeno, 230 m (QCAZ 28277, 28280, 28395); intersection of the Tarapoa-Puerto Carmen road and Río Cuyabeno, 290 m (QCAZ 28427); PROVINCIA ZAMORA CHINCHIPE: Centro Shuar Yawi, 940 m (QCAZ 31016, 31032–3, 31047, 31051, 31053).

Osteocephalus deridens.—ECUADOR: PROVINCIA ORELLANA: Estación Científica Yasuní, Universidad Católica del Ecuador, Parque Nacional Yasuní (QCAZ 12556).

Osteocephalus festae.—ECUADOR: PROVINCIA LOJA: San Francisco, Arco Iris Reserve, Parque Nacional Podocarpus (3.98845º S, 79.09298º W), 2200 m (QCAZ 39364); PROVINCIA MORONA SANTIAGO: Río Napinaza, 6.6 km N from General Leonidas Plaza (Limón) in the road to Mendez (2.92665º S, 78.40701º W), 1010 m (QCAZ 26283, 26304, 26488, 26552, 26561, 32835, 38081, 38420, 39799, 39804–6, 39798–803, 39808–12); San Carlos, San Miguel and Río Oro, 600–1200 m (QCAZ 11624–26); PROVINCIA ZAMORA CHINCHIPE: Miasí Alto (4.25025º S, 78.61740º W), 1250–1300 m (QCAZ 41039); Reserva Tapichalaca (4.55004º S, 79.12914º W), 1637 m (QCAZ 45674); PERU: REGIÓN DE AMAZONAS: PROVINCIA DE BAGUA: Cataratas de Paraiso-Chonza Alta (5.60264 S, 78.3985 W), 1342 m (CORBIDI 760, 761, 762, 763, 764, 758, 759); Camñopite (5.61469 S, 78.33192 W), 1650 m (CORBIDI 1962, 1963, 1964, 1965, 2992); REGIÓN DE SAN MARTÍN: PROVINCIA MARISCAL CACERES: Río Lejia (6.83655 S, 77.48603 W), 1500 m (CORBIDI 623, 624); PROVINCIA RIOJA: Bajo Naranjillo (5.81571, 77.33668 W), 844 m (CORBIDI 3386).

Osteocephalus fuscifacies.—ECUADOR: PROVINCIA NAPO: El Tena-Talag Road, 15 km from Tena, 550 m (QCAZ 8806); PROVINCIA ORELLANA: Pompeya-Iro Road, 38 km SE from Pompeya (QCAZ 8137); Estación Científica Yasuní, Universidad Católica del Ecuador, 240 m (QCAZ 20785).

Osteocephalus mutabor.—ECUADOR: PROVINCIA NAPO: Chontapuntas, Comunidad Sumak Sacha-Pozo Yuralpa Centro 1 (QCAZ 28646–48); Huino, around the waterfall (QCAZ 30916–17, 30919–20, 30922–23, 30925–26); PROVINCIA ORELLANA: km 22 Pompeya-Iro Road, 287 m (QCAZ 42999); PROVINCIA PASTAZA: Pomona, Fundación Hola Vida, 846 m (QCAZ 25603); Cantón Santa Clara, Río Pucayacu, Colonia Mariscal Sucre (QCAZ 36935, 36946, 40253); PROVINCIA SUCUMBIOS: Puerto Bolívar, 240 m (QCAZ 28223).

Osteocephalus planiceps.—ECUADOR: PROVINCIA DE NAPO: Chontapuntas, Comunidad Sumak Sacha-Pozo Yuralpa Centro 1 (QCAZ 28648); PROVINCIA ORELLANA: Parque Nacional Yasuní, km 38 Pompeya-Iro Road, 280 m (QCAZ 5134, 14842); Estación Científica Yasuní, Universidad Católica del Ecuador, 240 m (QCAZ 14844, 20797–800); PROVINCIA SUCUMBIOS: La Selva lodge, 250 m (QCAZ 7408, 12093–95).

Osteocephalus taurinus.—ECUADOR: PROVINCIA ORELLANA: Parque Nacional Yasuní, km 97 Pompeya-Iro road, 450 m (QCAZ 5301); Estación Científica Yasuní, Universidad Católica del Ecuador, 220 m (QCAZ 9007, 10604, 14804, 14954, 24449–50); PROVINCIA SUCUMBÍOS: Reserva de Producción Faunística Cuyabeno, 220 m (QCAZ 5871–77); Puerto Bolívar, 240 m (QCAZ 27916, 27920); Zábalo, 220 m (QCAZ 27982, 28015); Chiritza-Puerto El Carmen road, intersection with Río Aguas Negras, 270 m (QCAZ 28485); Tarapoa-Puerto El Carmen road, intersection with Río Cuyabeno, 290 m (QCAZ 28435–36); PROVINCIA ZAMORA CHINCHIPE: Shaime, Nangaritza, 980 m (QCAZ 18230).

Osteocephalus verruciger.—ECUADOR: PROVINCIA MORONA SANTIAGO: Nueve de Octubre (QCAZ 32266); Bosque Protector Abanico, Morona (EPN-H 11444–45); Río Sardinayacu, Palora, Parque Nacional Sangay (EPN-H 5940–42, 5947); PROVINCIA NAPO: E of Volcán Sumaco, 1570 m (QCAZ 1560, 1562); Lago Agrio road between Cascabel 1 and 2, 1600 m (QCAZ 7783–84); Sumaco, 1800–2100 m (QCAZ 8964); Pacto Sumaco (QCAZ 10907); km 13 (Loreto-Coca road), 1324 m (QCAZ 22201); Río Hollín (QCAZ 1681, 2405); Coordillera de los Guacamayos, Cosanga-Archidona road, 1600 m (QCAZ 12206, 41108); El Reventador (QCAZ 29208); Cascada San Rafael, 1553 m (QCAZ 363, 13225,13247, 16954, 32032–36); Cosanga, 339 m (QCAZ 15942); PROVINCIA SUCUMBIOS: Quito-Lago Agrio road, Río Azuela, 1680 m (QCAZ 15149, 15991–97, 16220, 16953, 22497, EPN-H 6341, 11987, 12105–07, 12112, 12143); La Bonita (QCAZ 3175); trail to Volcán Reventador, Gonzalo Pizarro (EPN-H 7052–53, 7059–60).

Osteocephalus yasuni.—ECUADOR: PROVINCIA SUCUMBIOS: Zábalo, 220 m (QCAZ 27998); Playas de Cuyabeno, 230 m (QCAZ 27816).
